# 

**Published:** 1899-02

**Authors:** 


### Southern Medical Record

A Monthly Journal of Medicine and Surgery.

VOL. XXIX. ATLANTA, GA., FEBRUARY, 1899. No. 2.

BERNARD WOLFF, M. D., EDITOR.

ASSOCIATE EDITORS:

A. W. GRIGGS, M. D. — J. McFADDEN GASTON, M. D.

J. McFADDEN GASTON, Jr., M. D. — WILLIS F. WESTMORELAND, M. D

MICHAEL HOKE, M.D., BUSINESS MANAGER.

Entered at the Post-office at Atlanta, Ga., as Second-class Matter.

